# Effect of esketamine vs dexmedetomidine adjunct to propofol sedation for pediatric 3Tesla magnetic resonance imaging: a randomized, double-blind, controlled trial

**DOI:** 10.1186/s40001-022-00890-x

**Published:** 2022-11-21

**Authors:** Shang-xian Xu, Xi-sheng Shan, Jin-meng Gao, Hua-xian Liu, Wei-rong Chen, Shan-shan Gao, Fu-hai Ji, Ke Peng, Qian Wang

**Affiliations:** 1 Department of Anesthesiology, Children’s Hospital of Soochow University, 92 Zhongnan Steet, Suzhou, Jiangsu China; 2 Department of Anesthesiology, First Affiliated Hospital of Soochow University, 188 Shizi Street, Suzhou, 215006 Jiangsu China; 3 Institute of Anesthesiology, Soochow University, Suzhou, Jiangsu China

**Keywords:** Esketamine, Dexmedetomidine, Propofol, Pediatric sedation, 3 T MRI

## Abstract

**Background:**

Adequate sedation is essential for pediatric patients undergoing 3Tesla (T) magnetic resonance imaging (MRI). Using propofol alone is associated with patient arousing and adverse airway events. This study aimed to assess esketamine vs dexmedetomidine adjunct to propofol sedation for pediatric 3 T MRI.

**Methods:**

In this randomized, double-blind, controlled trial, 114 pediatric patients aged between 6 months and 8 years were randomly assigned, in a 1:1 ratio, to the esketamine–propofol group or the dexmedetomidine–propofol group. Sedation was provided with esketamine or dexmedetomidine in combination with propofol titration. The primary outcome was the total dose of propofol. Secondary outcomes included propofol infusion dose, adverse events, time to emergence from sedation, and time to discharge from recovery room.

**Results:**

A total of 111 patients completed this study (56 in the esketamine–propofol group and 55 in the dexmedetomidine–propofol group). All MRI procedures were successfully performed under sedation. The total median (IQR) dose of propofol was significantly lower in the esketamine–propofol group (159.8 [121.7, 245.2] μg/kg/min) than that in the dexmedetomidine–propofol group (219.3 [188.6, 314.8] μg/kg/min) (difference in medians [95% CI] =  − 66.9 [− 87.8 to − 43.0] μg/kg/min, *P* < 0.0001). The use of esketamine resulted in a lower dose of propofol for titration (difference in medians [95% CI] =  − 64.3 [− 75.9 to − 51.9] μg/kg/min), a shorter time to emergence (difference in means [95% CI] =  − 9.4 [− 11.4 to − 7.4] min), and a reduced time to recovery room discharge (difference in means [95% CI] =  − 10.1 [− 12.1 to − 8.2] min). In the dexmedetomidine–propofol group, 2 patients experienced upper airway obstruction and 6 patients had bradycardia. No episodes of oxygen desaturation or other adverse events were observed.

**Conclusions:**

Although both regimens provided effective sedation for pediatric 3 T MRI, the esketamine–propofol sedation reduced propofol requirement and facilitated recovery, without detection of increased adverse effects in the studied population.

*Trial registration* Chinese Clinical Trial Registry (identifier: ChiCTR2100048477).

## What is known

- Adequate sedation is essential for pediatric patients undergoing MRI.
- Using propofol alone is associated with patient arousing and adverse airway events.

## What is new

- MRI procedures can be successfully performed under sedation with esketamine–propofol or dexmedetomidine–propofol.
- Esketamine–propofol sedation reduced propofol requirement and facilitated recovery, without significant adverse events.

## Introduction

Magnetic resonance imaging (MRI) is widely used for diagnostic imaging of many pediatric diseases [1, 2]. Compared with early MRI with field strength of 1.5 Tesla (T), 3 T MRI with better image quality and spatial resolution has become the clinical standard. However, 3 T MRI scans produce high noise in excess of 130 decibels, twice that of 1.5 T scans [3]. The noise and vibration during MRI can cause children’s arousal and body movement, which leads to poor image quality and MRI interruptions. To remain motionless and acquire high quality images, adequate sedation is essential in children, particularly younger children.

Propofol is widely used for sedation in various procedures. Propofol has a narrow therapeutic window for children [4, 5]. Increased or repeated doses of propofol may lead to deep sedation, loss of airway protective reflexes, apnea, upper airway obstruction, oxygen desaturation, and hypotension [2, 6–8]. Dexmedetomidine is a highly selective α-2 receptor agonist that offers sedative, analgesic, and anxiolytic effects, with minimal respiratory depression and a relatively short elimination half-life [9]. Owing to its efficacy and safety profile, dexmedetomidine has gained increasing popularity in pediatric patients as an adjuvant to propofol for imaging examinations. Studies have shown that dexmedetomidine in combination with propofol for MRI in children decreased propofol requirements, increased sedation success rate, and reduced sedation-related adverse effects when compared to propofol alone, but may increase the recovery time and the incidence of bradycardia [2, 8, 10]. A recent study demonstrated the trends of anesthetic practice of 24,052 MRI scans over a 7-year period and showed that the increased use of propofol–dexmedetomidine combination offered a smoother sedative technique but prolonged the post anesthesia care unit stays [11]. The 5 most common sedation techniques included propofol only, propofol combined with dexmedetomidine, propofol combined with other adjuncts (ketamine, midazolam, or fentanyl), volatile combined with propofol, and volatile only. Compared with propofol only, the use of volatiles increased the risk of hypotension, bradycardia, and hypoxia; the use of propofol combined with other adjuncts increased the incidence of severe hypoxia [11].

Ketamine which produces analgesia, sedation, and stable hemodynamics and respiratory function is another useful adjuvant to propofol sedation for pediatric procedures [12–15]. The use of ketamine is limited by its adverse effects, such as nausea and vomiting, laryngospasm, and psychotomimetic effects [16, 17]. Esketamine is an S-enantiomer that has twofold higher sedative potency and fewer side effects compared with racemic ketamine [18]. A recent study showed that esketamine effectively countered opioid-induced respiratory depression [19]. Thus, esketamine could be an attractive adjuvant to propofol sedation for pediatric MRI procedures.

In this study, we aimed to investigate the effects of esketamine in combination with propofol, compared to the dexmedetomidine–propofol combination, for sedation in pediatric patients undergoing 3 T MRI examinations. We hypothesized that a low-dose esketamine adjunct to propofol sedation would decrease propofol requirements, reduce respiratory and hemodynamic events, and facilitate post-procedure recovery.

## Methods

### Ethics and Registration

The study protocol was approval by the Ethics Committee of Children’s Hospital of Soochow University (No. 2021009) on June 10, 2021. This study was prospectively registered on the Chinese Clinical Trial Registry (Identifier: ChiCTR2100048477) on July 9, 2021. The study was conducted in accordance with the Declaration of Helsinki. We obtained written informed consent from the parents or guardians of all participants in this study. All patients could decline participation or request withdrawal from the study at any time, without the need to give specific reasons.

### Study design

This researcher-initiated, single-center, prospective, randomized, double-blind, controlled trial was carried out at the Children’s Hospital of Soochow University from July 12, 2021 to August 30, 2021. The Children’s Hospital of Soochow University is a referral medical center, where the MRI procedures are performed in approximately 2000 pediatric patients each year. This report follows the Consolidated Standards of Reporting Trials (CONSORT) Statement [20].

### Study patients

Pediatric patients aged between 6 months and 8 years with American Society of Anesthesiologists (ASA) Status Classification I or II, scheduled for 3 T MRI procedures under sedation were eligible for participation. The exclusion criteria were: (1) hemodynamic or respiratory instability (dehydration, shock, hypoadrenalism, hyperadrenalism, respiratory depression, or acute respiratory failure), (2) congenital heart disease with right-to-left shun, (3) increased intracranial or intraocular pressure, (4) cognitive impairment, or behavioral or psychological disorders, (5) history of more than three times of general anesthesia, and (6) allergies to the medications used in this study.

### Randomization and blinding

An independent research assistant performed the randomization with the use of an online tool (https://www.sealedenvelope.com/randomisation/) to allocate patients into either the esketamine–propofol group or the dexmedetomidine–propofol group. The randomization was generated with an allocation ratio of 1:1 and permuted block sizes of 2 and 4. The allocation was concealed using sealed opaque envelopes. The investigators were unaware of the details of randomization. According to the randomization results, an independent research nurse who did not participate in the subsequent study prepared the study medications: esketamine diluted with normal saline to a final concentration of 0.5 mg/ml, and dexmedetomidine to 1 μg/ml. This nurse did not have contacts with the investigators. Both esketamine and dexmedetomidine were clear and colorless fluids, and they were kept in identical syringes with the labels of patient number. Thus, it was impossible to distinguish them. All patients, peri-procedure care providers, and post-procedure observers were all blinded to the group assignment.

### Sedation for MRI procedures

All patients were fasted for 2 h for clear liquids, 4 h for breast milk, and 6 h for infant formula, nonhuman milk and light meal [21]. In a waiting area, the baseline heart rate (HR), systolic blood pressure (SBP), and diastolic blood pressure (DBP) were recorded before intravenous cannula insertion. Approximately 1 h after the use of a skin-numbing local anesthetic (Compound Lidocaine Cream), the cannulation was finished by a skilled nurse when the children were watching cartoon movies and accompanied by their parents, which minimized the fear of needles. After that, patients were transferred to the MRI room accompanied by their parents.

Supplemental oxygen at a flow of 1 L/min was delivered via nasal cannula. Throughout the study, HR, SBP, DBP, and peripheral oxygen saturation (SpO_2_) were monitored. After sedation induction, patients were positioned with a soft roll under the neck and shoulders. Upon the completion of MRI, the propofol infusion was stopped and the patients were transferred to a recovery room (RR). Recovery was assessed using the modified Aldrete score at 5 min intervals, and a score ≥ 9 indicated readiness for RR discharge [22–24]. To ensure consistency and reduce potential bias, the perioperative care for patients in this study was provided by the same multidisciplinary team.

Episodes of upper airway obstruction such as snoring, stridor, pharyngeal obstruction, and laryngospasm were managed by airway maneuvers (jaw thrust or chin lift). Oxygen desaturation was defined as SpO_2_ < 94% [25]. If the obstruction or desaturation was unresolved, patients received increased concentration of inspired oxygen and airway interventions (oropharyngeal airway, face mask ventilation, laryngeal mask airway, tracheal intubation, and positive-pressure ventilatory assistance). Hemodynamic data including HR, SBP, and DBP were recorded at baseline, immediately after induction, 5 min after the beginning of MRI, the end of MRI examinations, 5 min in the RR, and at the time of RR discharge. Bradycardia was defined as a decrease of HR > 20% from baseline [26, 27]. Severe bradycardia (defined as a decrease of HR > 30% from baseline) was treated with atropine 0.1 mg/kg. Hypotension (defined as a decrease of SBP > 20% from baseline) [26, 27] was treated with fluid administration and/or ephedrine based on the discretion of the anesthesiologist.

### Study interventions

An attending anesthesiologist performed the sedation procedure consisting of an induction phase and a titration phase, which was standardized for all patients in this study. For induction of sedation, a loading dose of propofol 1.5 mg/kg was administered to all patients. After that, the esketamine–propofol group received esketamine 0.15 mg/kg intravenously over 3 min, and the dexmedetomidine–propofol group received dexmedetomidine 0.3 μg/kg intravenously over 3 min [7, 28, 29]. We administered propofol prior to study drugs to induce a prompt sedation status in children, as propofol has a fast onset profile.

At the commencement of MRI, the target level of sedation was a Ramsey sedation score (RSS) of 6 (no response to a light glabellar tap or loud auditory stimulus) [30]. During the scanning, the anesthesiologist continuously monitored the level of sedation with the RSS target of 5–6 (a sluggish or no response to loud auditory stimulus). The target level of sedation was achieved using propofol titration. Propofol was infused at a rate of 50–300 μg/kg/min using an infusion system suitable for MRI, and boluses of 0.3–0.5 mg/kg could be administered at the discretion of the anesthesiologist. If the anesthesiologist noticed a deep sedation with signs of airway obstruction, hypotension, or bradycardia, the infusion of propofol was stopped. Inadequate sedation was defined as that the MRI procedures could not be completed due to body movement during the scanning. If inadequate sedation occurred, patients would receive general anesthesia with laryngeal mask using remifentanil, sevoflurane, and rocuronium to complete the MRI examination.

### Primary and secondary outcomes

The primary outcome of this study was the total dose of propofol (expressed as μg/kg/min), defined as the loading dose (during the induction phase) plus the continuous infusion dose and the boluses (during the titration phase).

The secondary outcomes included the dose of propofol for titration (continuous infusion dose and boluses), the incidences of adverse events (upper airway obstruction, oxygen desaturation, bradycardia, and bradycardia with intervention), time to emergence from sedation (defined as the time interval between discontinuing propofol infusion and reaching an RSS of 2), and time to RR discharge (defined as the time interval between discontinuing propofol infusion and discharge from RR).

### Peri-procedure and follow-up data

The peri-procedure data included hemodynamic changes, RSS scores, excessive salivation, hypotension, laryngospasm, scanning time, radiologist satisfaction scores, nausea and vomiting, the incidence of emergence delirium, and the pediatric anesthesia emergence delirium (PAED) scores. Emergence delirium was assessed using the PAED scores at emergence, 15 min after emergence, and RR discharge. The PAED consists of 5 dimensions: eye contact, purposeful actions, awareness of surroundings, restlessness, and inconsolability (a score of 0–4 for each item). A score of PAED ≥ 10 indicates the occurrence of emergence delirium [31–33]. The 24 h follow-up data were collected via telephone, including poor appetite, nausea and vomiting, and parent satisfaction scores. Radiologist and parent satisfactions were assessed using a numerical rating scale (NRS) of 0–10 (0 = not satisfied; 10 = very satisfied).

### Sample size calculation

In our pilot observation using dexmedetomidine–propofol sedation for 30 children undergoing 3 T MRI (unpublished data), the mean total dose of propofol was 232.3 μg/kg/min with the standard deviation (SD) of 91.3 μg/kg/min. A recent study showed that a low-dose esketamine reduced the total dose of propofol by 21% for sedation in endoscopic procedures [29]. Based on these, we hypothesized that the use of esketamine would reduce the total dose of propofol by 20% in our patients. With an α = 0.05, a power = 80%, and a possible dropout rate of 10%, a total of 114 patients were planned in this study (*n* = 57 in each arm). The number of patients required in this study was calculated using the PASS software (version 11.0.7; NCSS, LCC, Kaysville, UT).

### Statistical analysis

Continuous variables were checked for normal distribution using the Kolmogorov–Smirnov test. Normally distributed data are presented as mean ± SD and analyzed using the independent Student’s t test or repeated measures analysis of variance followed by Dunnett or Sidak test, as appropriate. Skewed data are presented as median (interquartile range, IQR) and analyzed using the Mann–Whitney *U* test. Categorical data are presented as number (%) and analyzed using chi-square test or Fisher’s exact test.

For the primary outcome, a two-sided *P* value < 0.05 denotes a statistically significant difference. For the 7 secondary outcomes, multiple testing corrections were applied using the Bonferroni method, with a *P* value < 0.007 (i.e., 0.05/7) indicating a statistically significant difference. To assess the between-group differences, the effect size was analyzed using difference in means for normally distributed data, difference in medians for skewed data, or attributable risk for categorical data, with their 95% confidence intervals (CIs). Difference in medians and the 95% CIs were estimated using Hodges–Lehman estimation of location shift.

All analyses were done on the intention-to-treat basis. No interim analysis was planned. As we expected that missing data would be rare in our data set, we did not have plan for missing data imputation. Statistical analyses were performed using the GraphPad Prism software (version 9.00; GraphPad, San Diego, CA).

## Results

### Study flow

**Fig. 1:**
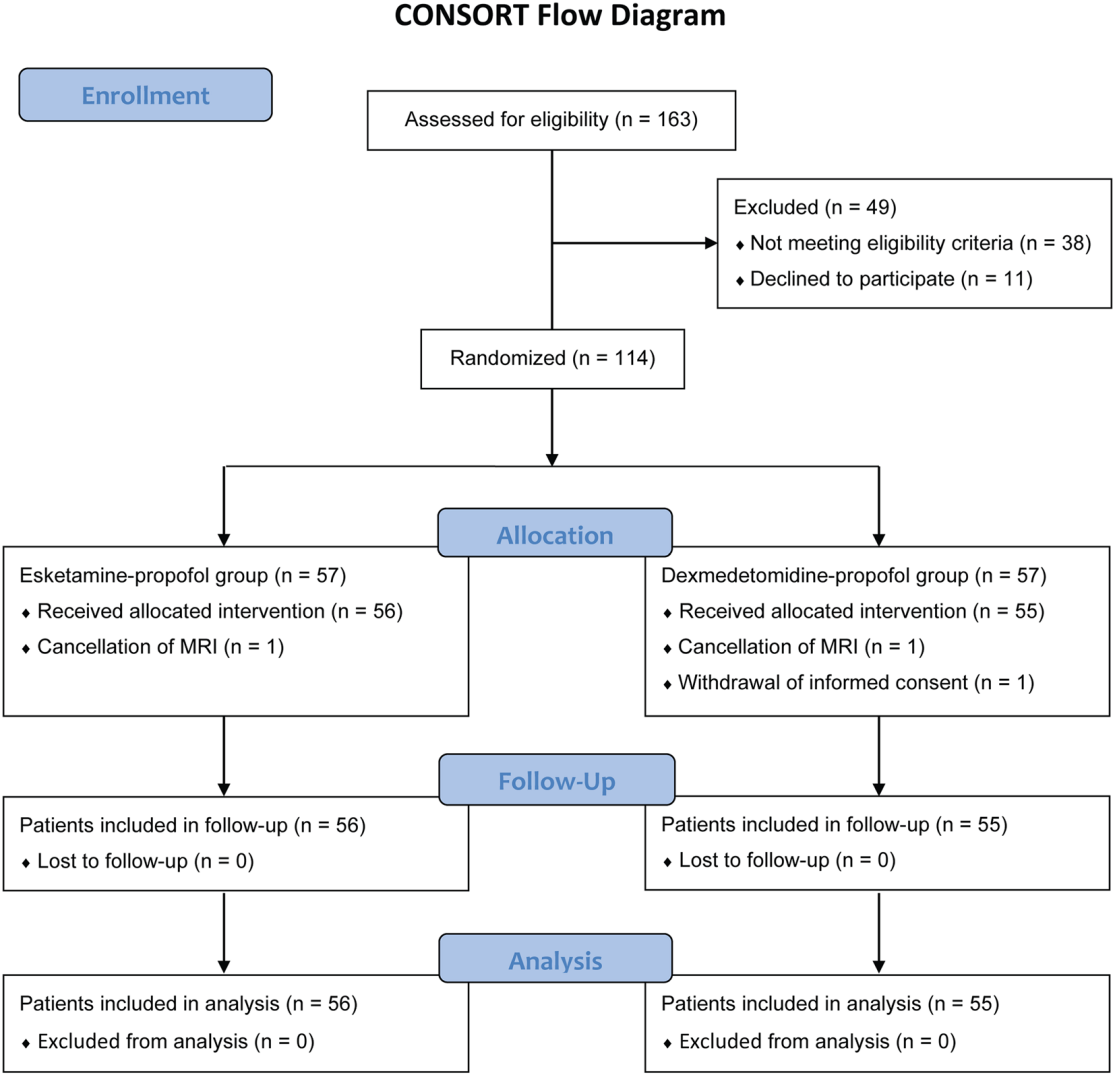
Study flowchart

Of 163 pediatric patients screened, 49 were excluded (38 did not meet the eligibility criteria and 11 declined to participate). Thus, 114 patients were randomly assigned to the esketamine–propofol group or the dexmedetomidine–propofol group. After randomization, 3 patients were excluded due to cancellation of MRI (*n* = 2) and withdrawal of informed consent (*n* = 1). Finally, a total of 111 patients (56 in the esketamine–propofol group and 55 in the dexmedetomidine–propofol group) completed this study (Fig. 1).

### Baseline characteristics

The two groups were comparable in terms of demographic data and baseline characteristics (Table 1). Children in the dexmedetomidine–propofol group were older (median age = 31 months) than those in the esketamine–propofol group (median age = 23 months). The median weight was 12–13.5 kg in the esketamine–propofol and dexmedetomidine–propofol groups, respectively. The major MRI sites were head (39.3 vs 49.1%) and spine (46.4 vs 38.2%).

**Table 1:** Demographics and baseline characteristics

	Esketamine–propofol (*n* = 56)	Dexmedetomidine–propofol (*n* = 55)
Age, month	23 (14.5, 37.5)	31 (19, 48)
Sex		
Male	31 (55.4%)	31 (56.4%)
Female	25 (44.6%)	24 (43.6%)
Weight, kg	12 (10, 15)	13.5 (10, 16)
ASA status		
I	41 (73.2%)	38 (69.1%)
II	15 (26.8%)	17 (30.9%)
Baseline measurement		
HR, beats/min	109 ± 9	108 ± 8
SBP, mmHg	93 (90, 98)	92 (89, 95)
DBP, mmHg	51 (48, 56)	52 (48, 56)
SpO_2_, %	99 (98, 99)	99 (98, 99)
Mild cough	5 (8.9%)	4 (7.3%)
Diagnostic category		
Endocrine	3 (5.4%)	7 (12.7%)
ENT/Ophthalmology	3 (5.4%)	4 (7.3%)
Hematology	1 (1.8%)	2 (3.6%)
Neurology	6 (10.7%)	6 (10.9%)
Neurosurgery	34 (60.7%)	27 (49.1%)
Orthopedics	3 (5.4%)	4 (7.3%)
Others	6 (10.7%)	5 (9.1%)
MRI site		
Head	22 (39.3%)	27 (49.1%)
Spine	26 (46.4%)	21 (38.2%)
Trunk	1 (1.8%)	3 (5.5%)
Limbs	2 (3.6%)	2 (3.6%)
Head + spine	3 (5.4%)	2 (3.6%)
Head + trunk	2 (3.6%)	0 (0%)

Data are mean ± standard deviation, median (interquartile range), or number (%)

*ASA* American Society of Anesthesiologists, *HR* heart rate, *SBP* systolic blood pressure, *DBP* diastolic blood pressure, *SpO*_*2*_ peripheral oxygen saturation, *MRI* magnetic resonance imaging

### Primary and secondary outcomes

Table 2 shows the primary and secondary outcomes. The median (IQR) total dose of propofol was 159.8 (121.7, 245.2) μg/kg/min in the esketamine–propofol group compared with 219.3 (188.6, 314.8) μg/kg/min in the dexmedetomidine–propofol group (difference in medians =  − 66.9 μg/kg/min, 95%CI =  − 87.8 to − 43.0 μg/kg/min, *P* < 0.0001).

**Table 2:** Primary and secondary outcomes

	Esketamine–propofol (*n* = 56)	Dexmedetomidine–propofol (*n* = 55)	Effect size (95% confidence interval)^a^	*P* value
Primary outcome^b^				
Total dose of propofol, μg/kg/min	159.8 (121.7, 245.2)	219.3 (188.6, 314.8)	− 66.9 (− 87.8 to − 43.0)	< 0.0001
Secondary outcomes^c^				
Dose of propofol for titration, μg/kg/min	91.0 (78.8, 134.0)	155.6 (138.8, 193.3)	− 64.3 (− 75.9 to − 51.9)	< 0.0001
Upper airway obstruction	0 (0%)	2 (3.6%)	− 0.04 (− 0.12 to 0.06)	0.243
Oxygen desaturation	0 (0%)	0 (0%)	0.00 (− 0.08 to 0.08)	> 0.999
Bradycardia	0 (0%)	6 (10.9%)	− 0.11 (− 0.21 to 0.01)	0.013
Bradycardia with intervention	0 (0%)	2 (3.6%)	− 0.04 (− 0.12 to 0.06)	0.243
Time to emergence, min	11.2 ± 5.1	20.6 ± 5.4	− 9.4 (− 11.4 to − 7.4)	< 0.0001
Time to discharge, min	21.6 ± 5.4	31.7 ± 4.9	− 10.1 (− 12.1 to − 8.2)	< 0.0001

Data are mean ± standard deviation, median (interquartile range), or number (%)

^a^Effect size is reported as difference in means for normally distributed data, difference in medians for skewed data, or attributable risk for categorical data

^b^For the primary outcome, *P* < 0.05 indicates a statistically significant difference

^c^For the secondary outcomes, *P* < 0.007 indicates a statistically significant difference after multiple testing corrections using the Bonferroni method

For the secondary outcomes, the esketamine–propofol group required a lower infusion dose of propofol (difference in medians =  − 64.3 μg/kg/min, 95%CI =  − 75.9 to − 51.9 μg/kg/min, *P* < 0.0001), a reduced time to emergence (difference in means =  − 9.4 min, 95%CI =  − 11.4 to − 7.4 min, *P* < 0.0001), and a shorter time to RR discharge (difference in in means =  − 10.1 min, 95%CI =  − 12.1 to − 8.2 min, *P* < 0.0001). No patient had bradycardia in the esketamine–propofol group, whereas 6 patients developed bradycardia in the dexmedetomidine–propofol group (attributable risk =  − 0.11, 95%CI =  − 0.21 to 0.01, *P* = 0.013; not statistically different after multiple testing correction). Two patients in the dexmedetomidine–propofol group experienced upper airway obstruction which was resolved by jaw thrust and chin lift. Oxygen desaturation was not observed in either group, and no patient needed increased concentration of inspired oxygen or airway interventions.

### Peri-procedure and follow-up data

**Fig. 2:**
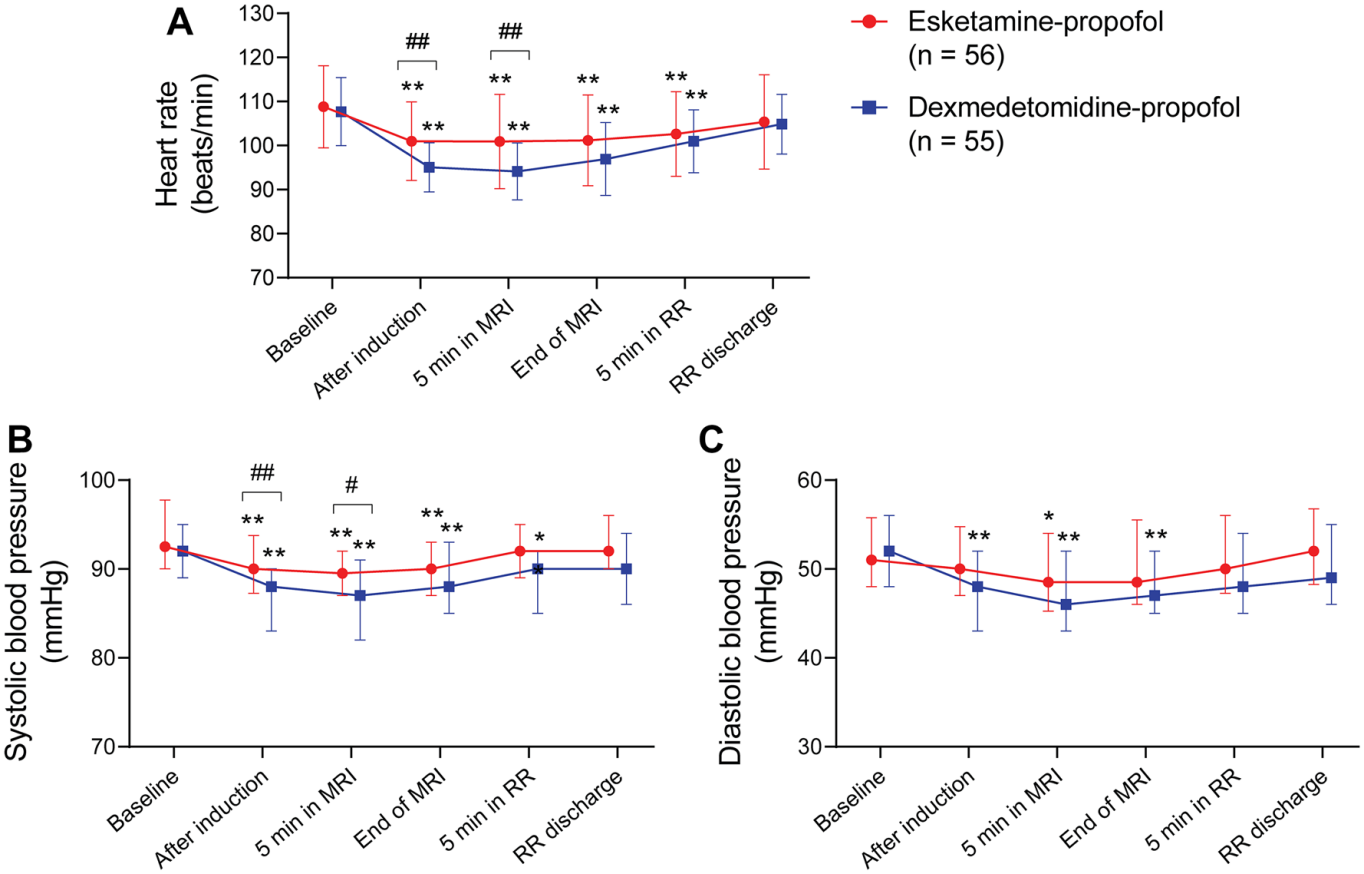
Hemodynamic changes throughout the study. **A** Heart rate. Data are mean ± standard deviation. **B** Systolic blood pressure. Data are median (interquartile range). **C** Diastolic blood pressure. Data are median (interquartile range). *P < 0.05, **P < 0.01 vs. the baseline value; ^#^*P* < 0.05, ^##^*P* < 0.01 for the comparisons shown

The HR values were significantly lower at several timepoints (from immediately after induction to 5 min in the RR) than that at baseline in both groups (Fig. 2A). The esketamine–propofol group had higher HR compared to the dexmedetomidine–propofol group after induction (101 ± 9 vs 95 ± 6 beats/min) and at 5 min after the beginning of MRI (101 ± 11 vs 94 ± 6 beats/min). Both groups showed decreased SBP and DBP after induction and during the procedures (Fig. 2B, C). The median SBP values were higher in the esketamine–propofol group immediately after induction (90 vs 88 mmHg) and at 5 min after the beginning of MRI (90 vs 87 mmHg).

Table 3 presents the peri-procedure and follow-up data. The RSS results showed that the two groups had comparable sedation levels at the beginning of MRI, 5 min in MRI, and the end of MRI. No patient experienced inadequate sedation and body movement that interrupted the MRI scanning. Two patients in esketamine–propofol group showed excessive salivation after induction, which did not lead to consequences such as coughing or respiratory events and no intervention was needed. There were no hypotension or laryngospasm events during the procedures. The median (IQR) scanning time was 22 (13.3, 35.8) and 21 (14, 35) min in the esketamine–propofol and dexmedetomidine–propofol groups, respectively. The radiologists reported comparable satisfaction scores for the two sedation regimens. No emergence delirium was observed in the RR, and there were no significant differences in the PAED scores during the observation. One patient in the esketamine–propofol group had nausea and vomiting in the RR. All patients completed the 24 h follow-up via telephone. Two patients in the esketamine–propofol group had poor appetite when having the first meal at approximately 2 h after the procedures. The median parent satisfactions score was 9 points in both groups.

**Table 3:** Peri-procedure and follow-up data

	Esketamine–propofol (*n* = 56)	Dexmedetomidine–propofol (*n* = 55)	*P* value
During the procedures			
RSS at beginning of MRI	6 (6, 6)	6 (6, 6)	> 0.999
RSS at 5 min in MRI	6 (5, 6)	6 (5, 6)	0.915
RSS at end of MRI	5 (5, 6)	5 (5, 6)	0.422
Inadequate sedation	0 (0%)	0 (0%)	> 0.999
Excessive salivation	2 (3.6%)	0 (0%)	0.496
Hypotension	0 (0%)	0 (0%)	> 0.999
Laryngospasm	0 (0%)	0 (0%)	> 0.999
Scanning time, min	22 (13.3, 35.8)	21 (14, 35)	0.643
Radiologist satisfaction scores	8 (8, 9)	9 (8, 9)	0.860
In the RR			
Emergence delirium	0 (0%)	0 (0%)	> 0.999
PAED scores at emergence	5 (5, 7)	5 (5, 6)	0.223
PAED scores at 15 min after emergence	3 (2, 3)	3 (2, 4)	0.325
PAED scores at RR discharge	2 (1, 3)	2 (2, 3)	0.836
Nausea and vomiting	1 (1.8%)	0 (0%)	> 0.999
24 h after the procedures			
Poor appetite	2 (3.6%)	0 (0%)	0.496
Nausea and vomiting	0 (0%)	0 (0%)	> 0.999
Parent satisfaction scores	9 (8, 9)	9 (7, 9)	0.465

Data are numbers (%) or medians (interquartile range)

*RSS* Ramsey sedation score, *PAED* Pediatric Anesthesia Emergence Delirium, *RR* recovery room

## Discussion

This randomized controlled trial of 111 pediatric patients undergoing 3 T MRI suggested that esketamine at a low dose of 0.15 mg/kg in combination with propofol significantly reduced the total propofol consumption (a relative reduction of ~ 27%) when compared to dexmedetomidine 0.3 μg/kg in combination with propofol. In addition, the use of esketamine led to a reduced time to emergence from sedation and a reduced time to RR discharge. Two patients experienced upper airway obstruction and 6 patients had bradycardia, all from the dexmedetomidine–propofol group. There were no episodes of oxygen desaturation or emergence delirium.

Previous studies showed that the dexmedetomidine–propofol combination provided adequate sedation for children undergoing MRI [2, 8]. Nagoshi and colleagues showed that a single dose of dexmedetomidine 0.5 μg/kg an adjuvant decreased the propofol requirement for sedation in MRI procedures (> 60% were 1.5 T MRI) [8]. Boriosi and colleagues reported increased patient arousal and respiratory events in children receiving propofol as the sole sedative after the transition of MRI from 1.5 T to 3 T in their institution [2]. To solve this problem, they used dexmedetomidine infusion of 1–2 μg/kg over 5–10 min prior to propofol administration. Their results showed that the dexmedetomidine–propofol sedation suppressed patient arousal and reduced total adverse events (particularly upper airway obstruction) but increased the discharge time [2]. In our institution, we also found that the use of propofol alone may not provide an adequate level of sedation for pediatric 3 T MRI. Based on our clinical practice, we used a low-dose dexmedetomidine of 0.3 μg/kg as an adjuvant to propofol sedation for the purpose of increasing the successful sedation rate, avoiding a prolonged time to recovery, and reducing the risk of bradycardia. Nonetheless, 6 patients receiving dexmedetomidine still developed bradycardia in this study, and 2 of them needed intervention with atropine.

Schmitz et al. reported that a single dose of racemic ketamine reduced the total propofol dose by ~ 50% for sedation in pediatrics undergoing MRI [34]. That is a larger reduction in the propofol dose compared with our study (by ~ 27%), which can be attributable to the different control groups (propofol alone in that study vs propofol plus dexmedetomidine in our study). Esketamine has been used in pediatric patients in different clinical settings. Van de Bunt and colleagues reported that procedural sedation with esketamine was effective and safe for children undergoing hydrostatic reduction for ileocolic intussusception [35]. Another retrospective study suggested that esketamine sedation for manipulation of pediatric forearm fractures in the emergency department led to acceptable patient outcomes, without adverse events following esketamine administration [36]. Similar to our results, a recent randomized study showed that a low-dose esketamine adjunct to propofol sedation in adult patients undergoing endoscopic retrograde cholangiopancreatography reduced the total amount of propofol, without affecting recovery time, patient and endoscopist satisfaction, or cardiorespiratory adverse events [29]. A recent study showed that the intranasal use of dexmedetomidine and esketamine provided satisfactory sedation for anesthesia induction in children [37]. Esketamine (2 ml: 50 mg) costs 91 RMB (13.5 US dollars), which is cost-effective compared to 10 min in the RR. In China, the National Medical Products Administration has approved the use of esketamine for perioperative sedation and analgesia (approval no. H20193336). As far as we know, our present study is the first randomized trial to show the efficacy and safety of esketamine adjunct to propofol sedation in children who underwent 3 T MRI procedures.

Children in the dexmedetomidine–propofol group were older than those in the esketamine–propofol group. We do not believe that the difference in age could influence our primary and secondary outcomes. Nonetheless, this might be relevant for HR and blood pressure. The reference ranges of vital signs in Chinese children are as follows: HR, 99–155 beats/min for 12–23 months, 80–130 beats/min for 24–59 months, and 65–115 beats/min for 60–143 months; SBP, 75–110 mmHg for 12–23 months, 80–120 mmHg for 24–59 months, and 90–135 mmHg for 60–143 months [38]. For pediatric patients, bradycardia or hypotension can be defined as a decrease in HR or BP relative to baseline values [26, 27]. The esketamine–propofol group did not show bradycardia or bradycardia with intervention. In contrast, 6 patients in dexmedetomidine–propofol group had bradycardia, and 2 of them needed intravenous atropine. Neither group experienced hypotensive events. Two patients in the dexmedetomidine–propofol had upper airway obstruction, while no patient in the esketamine–propofol had this event. Although the between-group differences in these events were not statistically significant, our findings suggest that the use of esketamine may better maintain stable heart rate and respiration.

In our study, we utilized the RSS to assess the depth of sedation, and bispectral index (BIS) was not used. A recent study suggested that using the sedation rating scale was adequate and sufficient to measure the depth of sedation during gastrointestinal endoscopic procedures [29]. Next, studies showed that the administration of ketamine or esketamine significantly increased the BIS values, which reduced the ability of BIS in predicting sedation/anesthesia levels [39, 40]. Esketamine may be associated with psychotomimetic and cognitive adverse effects including visual disturbances, vertigo, drowsiness, mood instability, and changes in perception of surroundings, time, colors, sounds, and body [29]. However, it is difficult for young children to express their experience. Thus, we instead used the PAED scores to evaluate eye contact, purposeful actions, awareness of surroundings, restlessness, and inconsolability in our patients, and the results showed that no patients in either group had emergence delirium.

## Limitations

There are several limitations. First, we included children aged from 6 months to 8 years with ASA status I or II. Children outside this age range or with higher ASA status scheduled for MRI under sedation were underrepresented in this study. Our results could provide some reference and further studies are required. Second, the observed between-group difference in the total dose of propofol was in line with the sample size calculation, but this study was not powered to detect any differences in the adverse events. If the study was powered for detecting a reduction in the incidence of serious adverse events (from 10 to 5%), it would increase the sample size to 966 patients with α = 0.05, power = 80%, and a dropout rate of 10%. Third, end-tidal carbon dioxide (EtCO_2_) is a more sensitive measure of respiratory depression than SpO_2_ which is a late warning of hypoventilation [41, 42]. Hypoventilation could be detected earlier and more accurately if EtCO_2_ monitoring was applied in our patients. However, EtCO_2_ monitoring during sedation is not routine clinical practice in our institution, as well as in China. According to a recent national survey, the use of capnography during sedation for endoscopy ranged from 0 to 29% in different provinces [43]. Fourth, we did not assess dissociation in our patients. Dissociation is a common adverse effect of esketamine, especially in patients receiving a higher dose of esketamine. Next, we used a single dose of dexmedetomidine or esketamine. The optimal doses of these adjuvants to propofol sedation in pediatric MRI need further investigation. Last, as a single-center study with a relatively small number of patients, the generalizability of our findings should be corroborated in larger multicenter trials.

## Conclusions

This study suggested that a low-dose esketamine vs dexmedetomidine adjunct to propofol sedation for pediatric 3 T MRI decreased propofol consumption and shortened recovery, without incurring bradycardia or other significant adverse events.

## Data Availability

All data relevant to the study protocol is included as part of this manuscript. The prospective listing of the study on the Chinese Clinical Trial Registry can be found at http://www.chictr.org.cn/showproj.aspx?proj=129659.

## Abbreviations

ASA: American Society of Anesthesiologists
CONSORT: Consolidated Standards of Reporting Trials
CI: Confidence intervals
DBP: Diastolic blood pressure
HR: Heart rate
IQR: Interquartile range
MRI: Magnetic resonance imaging
NRS: Numerical rating scale
PAED: Pediatric anesthesia emergence delirium
RR: Recovery room
SBP: Systolic blood pressure
SD: Standard deviation
SpO_2_: Peripheral oxygen saturation
